# *ALDH2* gene G487A polymorphism and coronary artery disease: a meta‐analysis including 5644 participants

**DOI:** 10.1111/jcmm.13443

**Published:** 2017-12-26

**Authors:** Yan‐yan Li, Hui Wang, Jing‐jing Wu, Hyun Jun Kim, Xin‐xing Yang, Hong‐yu Geng, Ge Gong

**Affiliations:** ^1^ Department of Geriatrics First Affiliated Hospital of Nanjing Medical University Nanjing China; ^2^ Institute of Clinical Medicine First Affiliated Hospital of Nanjing Medical University Nanjing China; ^3^ Department of cardiology First Affiliated Hospital of Nanjing Medical University Nanjing China; ^4^ Department of Nephrology First Affiliated Hospital of Nanjing Medical University Nanjing China; ^5^ Department of Physiology University of Cincinnati Cincinnati USA; ^6^ Department of Geriatrics Nanjing General Hospital Nanjing China

**Keywords:** *ALDH2*, G487A, Glu504Lys, Polymorphism, coronary artery disease

## Abstract

Several studies indicate the mitochondrial *Aldehyde Dehydrogenase‐2 (ALDH2)* gene G487A polymorphism may be correlated with coronary artery disease (CAD) susceptibility, but a clear consensus has yet to be reached. To elucidate the relationship between the *ALDH2* gene G487A polymorphism and CAD within the Chinese population, a meta‐analysis of 5644 subjects from nine individual studies was performed. Pooled odds ratios (ORs) and their corresponding 95% confidence intervals were assessed using random or fixed‐effect models depending the heterogeneity existence or not. Our meta‐analysis found a significant association between *ALDH2* gene G487A polymorphism and CAD in the Chinese population under allele (OR: 1.830, 95% CI: 1.560–2.140, *P* = 1.36 × 10^−13^), recessive (OR: 1.920, 95% CI: 1.530–2.390, *P* = 1.20 × 10^−8^), dominant (OR: 1.593, 95% CI: 1.336–1.900, *P* = 2.22 × 10^−7^), homozygous (OR: 2.280, 95% CI: 1.810–2.870, *P* = 3.17 × 10^−12^) and heterozygous genetic models (OR: 3.330, 95% CI: 2.070–5.370, *P* = 7.81 × 10^−7^). The positive correlation between the *ALDH2* gene G487A polymorphism and CAD makes the mutation a strong candidate as a genetic risk marker for CAD. Through further analysis, we also found that A allele carriers of *ALDH2* gene G487A polymorphism may be particularly susceptible to CAD.

## Introduction

Coronary artery disease (CAD) is a disease that affects many middle‐aged and elderly people and is usually accompanied by hypertension, diabetes mellitus and dyslipidaemia. ALDH2 is a mitochondria enzyme distributed in the various tissues. CAD describes a group of cardiovascular diseases that collectively cause more deaths than any other disease globally. The confluence of genomic and environmental risk factors make CAD difficult to treat effectively, but research on the genomic basis of CAD, has tremendously deepened our understanding of this complex disease.

Mitochondrial aldehyde dehydrogenase‐2 (ALDH2) is an enzyme known primarily for its role in alcohol metabolism. Once ethanol is converted to acetaldehyde, ALDH2 facilitates its conversion to acetate. More interestingly, ALDH2 was also found to play significant role in cardiac protection in a range of settings. It was found to alleviate cardiac ischaemic injury by removing 4‐HNE, a highly reactive compound associated with oxidative stress 1 and contributes GTN biotransformation by converting it to 1, 2‐glycerin dinitrate 2. ALDH2 was also able to reverse the cardiac hypertrophy caused by chronic alcohol consumption and improve the systolic dysfunction of alcoholic cardiomyopathy 3.

Human *ALDH2,* located on 12q24, contains 13 exons. The G487A polymorphism causes the glutamate at position 504 to be replaced by a lysine residue on exon 12 (rs671). While the mutation frequency of this polymorphism is only 5% in Western populations, East Asian populations can have mutations frequencies ranging from 30% to 50% 4. The G487A mutation is associated with a significant decrease in enzymatic activity and reduced biological function 5.Regression analysis showed that the *ALDH2* gene G487A polymorphism was associated with decreased high‐density lipoprotein cholesterol (HDL‐C) levels. Taking into consideration data produced by Shaul and Mineo in 2004 indicating a 10% decrease in HDL‐C levels correlated with a 13% increase in CAD risk, the relationship between the gene polymorphism and CAD is a very real possibility 6.

Many experiments on the relationship between *ALDH2* gene G487A polymorphism and CAD have been conducted, but results remain inconsistent. Though Guo *et al*. 7 and Wang *et al*. 8 both found that the rs671 polymorphism of ALDH2 was associated with CAD in Han Chinese possibly by influencing HDL‐C levels and endothelial asymmetric dimethylarginine(ADMA) levels. Xia *et al*. 9 found no significant association between them.

To survey the current landscape of the research, we conducted a meta‐analysis of 2887 CAD patients and 2757 controls from nine separate studies to evaluate the relationship of *ALDH2* gene G487A polymorphism and CAD (Appendix S1).

## Materials and methods

### Publication search and inclusion criteria

The terms ‘*ALDH2’*, ‘*G487A’*, ‘coronary artery disease’ and ‘polymorphism’ were used to search PubMed, Wan Fang database, VIP database, China National Knowledge Infrastructure, China Biological Medicine Database, EMBASE and Web of Science. The last research was updated on 25 August 2017 and the publication years ranged from 2010 to 2014.

The studies were selected according to the following major criteria: (*i*) Evaluation of the relationship of CAD and *ALDH2* gene G487A polymorphism. (*ii*) Coronary angiography was used to diagnose CAD with diagnosis criteria defined as the stenosis of one coronary artery by at least 50%. (*iii*) Studies were case–control or officially published cohort studies. (*iv*) The control group genotype followed Hardy–Weinberg equilibrium (HWE).

### Data extraction

Data were abstracted in the light of a standardized protocol. Three investigators performed the current meta‐analysis: two searched out the individual studies duplicately, and the third one would resolve the disagreement between the two investigators. Those studies that violated the selection criteria were repeatedly published or provided insufficient data were excluded. If similar data sets were used in different papers by the same author group, the data were used only once. The data table listed should include the following items as publication year, ethnicity, region, the first author's name, genotyping method, matching criteria, genotype number and total number of cases and controls.

### Statistical analyses

Five genetic models were used in the current meta‐analysis: allele (A *versus* G), recessive (AA *versus* GG+GA), dominant (AA+GA *versus* GG), homozygous (AA *versus* GG) and heterozygous (GA *versus* GG) genetic models were used. The allele model is defined as A allele *versus* G allele. The relationship of *ALDH2 g*ene G487A polymorphism and CAD was compared using the odds ratios (ORs) and their corresponding 95% confidence intervals (CIs). The chi‐square‐based *Q*‐tests were used to calculate the heterogeneity among the studies with significance was set at *P* < 0.05 level 10. If heterogeneity was present among the different studies, the random‐effect model (DerSimonian and Laird method) would be adopted 11. Otherwise, the fixed‐effect model would be used (the Mantel–Haenszel method) 12. *Z*‐test was used to assess the pooled OR, and the significance was set at *P* < 0.05 level.

Fisher's exact test was used to evaluate the HWE with significance set at *P* < 0.05 level. A funnel plot was used to assess the potential publication bias. The Egger's linear regression test on the OR was used to evaluate the funnel plot symmetry, and the significance was set at *P* < 0.05 level 13. Stata 12.0 (StataCorp, College Station, TX, USA) was used to perform the statistical analyses.

## Results

### Studies and populations

Information was abstracted from a total of 2887 CAD cases and 2757 controls (Table 1) 7, 8, 9, 14, 15, 16, 17, 18, 19 (Appendix S2). Seventeen papers were acquired through the retrieval process, among which nine papers were selected for the current meta‐analysis. Among the eight rejected studies, two papers were reviews. One paper deviated from the HWE 20 while another lacked a control group 21. Four papers were unrelated to the *ALDH2* gene G487A polymorphism or CAD.

**Table 1:** Characteristics of the investigated studies of the association between the *ALDH2* gene G487A (Glu504Lys) polymorphism and CAD in the Chinese population

Author	Year	Region	Genotype	CAD			Control			Matching criteria	Sample size(CAD/control)
				GG	GA	AA	GG	GA	AA		
GuoYJ 7	2010	Hunan	PCR‐RFLP	219	171	27	291	135	22	Age, gender, ethnicity, PHAC, HL	417/448
Wang YF 8	2014	Liaoning	PCR‐LDR	324	259	96	367	258	61	Age, gender, ethnicity, BMI, TC, LDL‐C, BUN, Creatinine, Uric acid	679/686
Xu F 14	2011	Beijing, Shandong	PCR	291	227	28	372	165	9	Age, gender,ethnicity, TG, smoking	546/546
BianY 15	2010	Shandong	PCR	54	48	4	146	63	3	Age, gender, ethnicity, BMI, TG, TC, LDL‐C, smoking, PHAC	106/212
Cao L 16	2010	Shandong	PCR	92	53	24	93	47	11	Age, gender, ethnicity, BMI, TG, TC, SBP, DBP	169/151
Jiang AH 17	2011	Beijing	PCR	106	48	8	25	8	1	Gender, ethnicity	162/34
Tan Z 18	2013	Hebei	PCR	106	92	12	154	62	5	Age, gender, ethnicity, BMI, PHAC TG, TC, HDL‐C, LDL‐C	210/221
Xia G 9	2011	Shanghai	Taqman	253	202	35	240	173	20	Age, ethnicity, BMI, DB, HP, TG, TC, HDL‐C, LDL‐C	490/433
Zhao Q 19	2014	Hunan	PCR	57	44	7	19	6	1	Age, gender, ethnicity	108/26

CAD, coronary artery disease; HDL‐C, high‐density lipoprotein cholesterol; HP, hypertension; PCR‐LDR, Polymerase chain reaction‐ligase detection reaction; PCR‐RFLP, Polymerase chain reaction‐restriction fragment length polymorphism; Case–control study design was adopted in the above studies. PHAC, Positive history of alcohol consumption (%); HL, Hyperlipidaemia (%);TC, total cholesterol; TG, total triglycerides; SBP, systolic blood pressure; DBP, diastolic blood pressure; LDL‐C, low‐density lipoprotein cholesterol; HDL‐C, high‐density lipoprotein cholesterol; BMI, body mass index; DB, Diabetes; HP, hypertension.

### Pooled analyses

**Figure 1:**
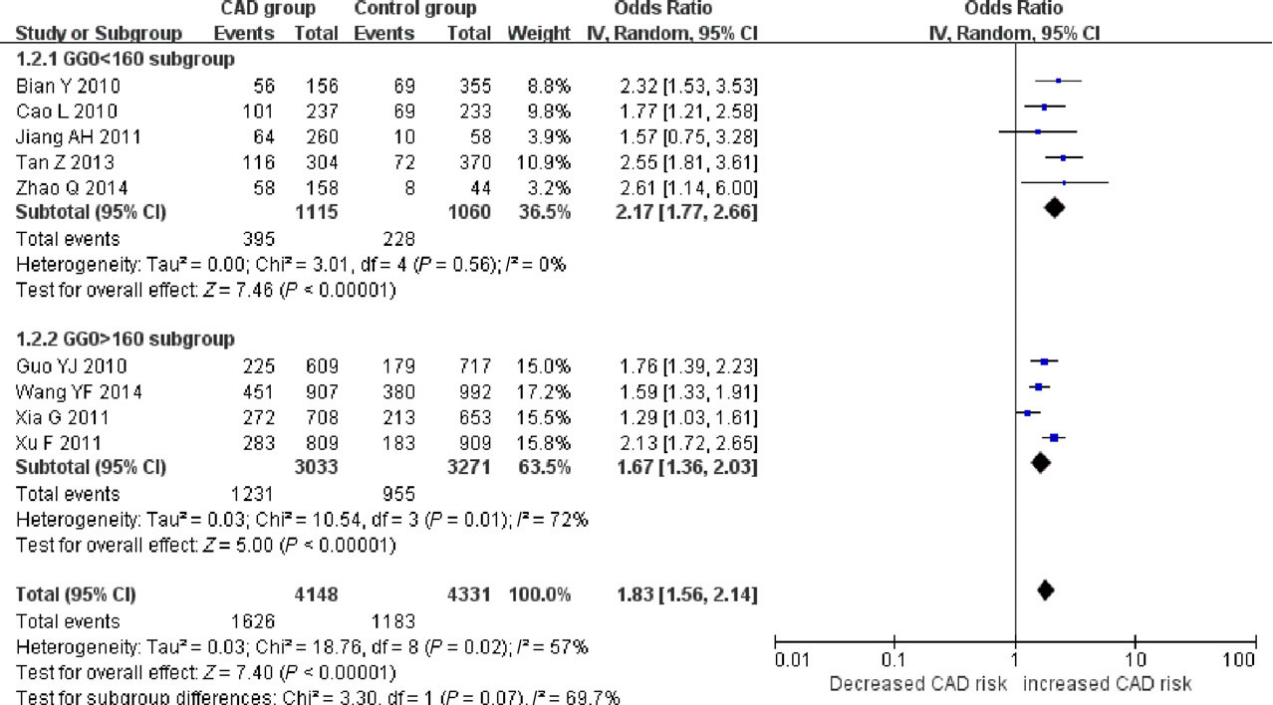
Forest plot of CAD associated with *ALDH2* gene G487A polymorphism under an allele genetic model (A *versus* G).

**Figure 2:**
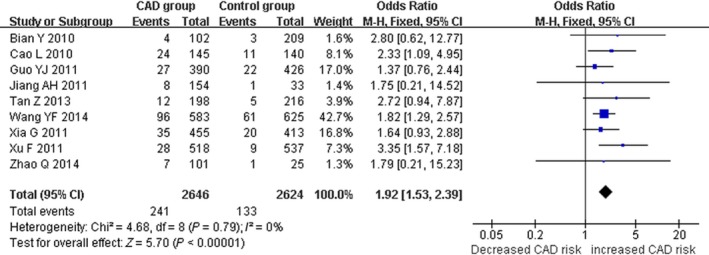
Forest plot of CAD associated with *ALDH2* gene G487A polymorphism under a recessive genetic model (AA *versus* GG+GA).

**Figure 3:**
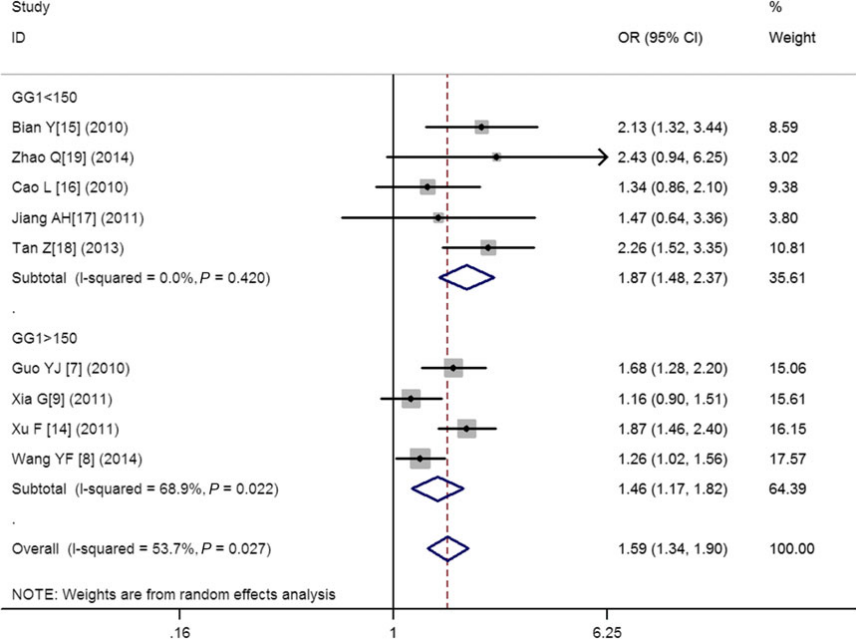
Forest plot of CAD associated with *ALDH2* gene G487A polymorphism under a dominant genetic model (AA+GA *versus* GG).

**Figure 4:**
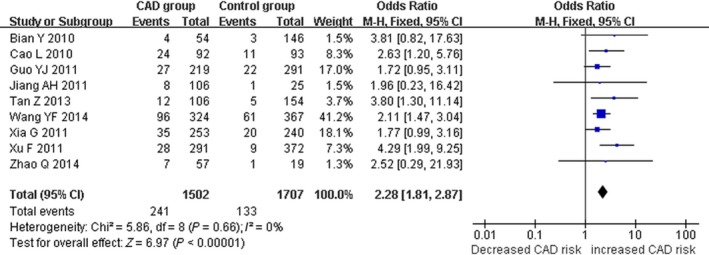
Forest plot of CAD associated with *ALDH2* gene G487A polymorphism under a homozygous genetic model (AA *versus* GG).

**Figure 5:**
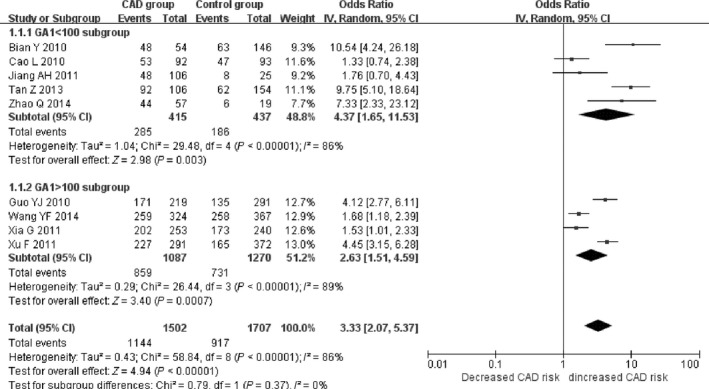
Forest plot of CAD associated with *ALDH2* gene G487A polymorphism under a heterozygous genetic model (GA *versus* GG).

There was a significant association between *ALDH2* gene G487A polymorphism and CAD in the Chinese population under allele (OR: 1.830, 95% CI: 1.560–2.140, *P* = 1.36 × 10^−13^), recessive (OR: 1.920, 95% CI: 1.530–2.390, *P* = 1.20 × 10^−8^), dominant (OR: 1.593, 95% CI: 1.336–1.900, *P* = 2.22 × 10^−7^), homozygous (OR: 2.280, 95% CI: 1.810–2.870, *P* = 3.17 × 10^−12^) and heterozygous genetic models (OR: 3.330, 95% CI:2.070–5.370, *P* = 7.81 × 10^−7^; Table 2; Figs 1, 2, 3, 4, 5).

**Table 2:** Summary of meta‐analysis of association between *ALDH2* gene G487A (Glu504Lys) polymorphism and CAD in the Chinese population

Genetic model	Pooled OR (95% CI)	*Z* value	*P* value	Study number	CAD size	control size	*P*_heterogeneity(*I*_^*2*^_*%*)_
Allele genetic model	1.830 (1.560–2.140)	7.40	1.36 × 10^−13^ a	9	2887	2757	0.02^a^ (57.0)
Subgroup 1: GG0 < 160	2.170 (1.770–2.660)	7.46	8.66 × 10^−14^ a	5	755	644	0.56 (0)
Subgroup 2: GG0 > 160	1.670 (1.360–2.030)	5.00	5.73 × 10^−7^ a	4	2132	2113	0.01^a^ (72.0)
Recessive genetic model	1.920 (1.530–2.390)	5.70	1.20 × 10^−8^ a	9	2887	2757	0.79 (0)
Dominant genetic model	1.593 (1.336–1.900)	5.18	2.22 × 10^−7^ a	9	2887	2757	0.027^a^ (53.7)
Subgroup 1: GG1 < 150	1.875 (1.485–2.367)	5.28	1.29 × 10^−7^ a	5	755	644	0.42 (0)
Subgroup 2: GG1 > 150	1.460 (1.171–1.821)	3.36	7.79 × 10^−4^ a	4	2132	2113	0.022^a^ (68.9)
Homozygous genetic model	2.280 (1.810–2.870)	6.97	3.17 × 10^−12^ a	9	2887	2757	0.66 (0)
Heterozygous genetic model	3.330 (2.070–5.370)	4.94	7.81 × 10^−7^ a	9	2887	2757	<0.00001^a^ (86.0)
Subgroup 1: GA1 < 100	4.370 (1.650–11.530)	2.98	0.003^a^	5	755	644	<0.00001^a^ (86.0)
Subgroup 2: GA1 > 100	2.630 (1.510–4.590)	3.40	0.0007^a^	4	2132	2113	<0.00001^a^ (89.0)

a *P* ≤ 0.05.

CAD, coronary artery disease; CI, confidence interval; OR, odds ratio; CAD size, the total number of CAD cases; control size, the total number of control group; Allele genetic model, total A allele *versus* total G allele; recessive genetic model, AA *versus* GG+GA; Dominant genetic model: AA+GA *versus* GG; Homozygous genetic mode, AA *versus* GG; Heterozygous genetic model, GA *versus* GG.

Under recessive and homozygous genetic models, no significant heterogeneity was detected in the Chinese population (*P*_heterogeneity_ > 0.05). There was significant heterogeneity, however, under allele, dominant and heterozygous genetic models (*P*_heterogeneity_ < 0.05). Meta‐regression was performed to find the sources of heterogeneity. Under the allele genetic model (*P*_heterogeneity_ = 0.02, *I* ^2^ = 57.0%), the GG genotype number in the control group (GG0) was detected to be the main heterogeneity source (*P* = 0.009). In the subgroup analysis stratified by GG0, no heterogeneity was found in the subgroup 1(GG0 < 160, *P*_heterogeneity_ = 0.56, *I* ^2^ = 0%). However, the heterogeneity still existed in subgroup 2 (GG0 > 160, *P*_heterogeneity_ = 0.01, *I* ^2^ = 72.0%). Under the dominant genetic model (*P*_heterogeneity_ = 0.027, *I* ^2^ = 53.7%), the GG genotype number in the CAD group (GG1) was confirmed to be the main heterogeneity source (*P* = 0.042). In the subgroup analysis stratified by GG1, there was no heterogeneity in the subgroup 1(GG1 < 150, *P*_heterogeneity_ = 0.42, *I* ^2^=0%). However, the heterogeneity still existed in subgroup 2 (GG1 > 150, *P*_heterogeneity_ = 0.022, *I* ^2^ = 68.9%). Under the heterozygous genetic model (*P*_heterogeneity_ < 0.00001, *I* ^2^ = 86.0%), the GA genotype number in the CAD group (GA1) was verified to be the main heterogeneity source (*P* = 0.002). In the subgroup analysis stratified by GA1, the heterogeneity still existed in both subgroups (subgroup 1, GA1 < 100, *P*_heterogeneity_ < 0.00001, *I* ^2^ = 86.0%; subgroup 2, GA1 > 100, *P*_heterogeneity_ < 0.00001, *I* ^2^ = 89.0%).

According to the three cutting point 160 for GG0, 150 for GG1, 100 for GA1, and two subgroups were divided. The subgroup 1 of smaller number group included five studies as Bian Y 15, Cao L 16, Jiang AH 17, Tan Z 18 and Zhao Q 19. The subgroup 2 of larger number group included four studies as Guo YJ 7, Wang YF 8, Xia G 9 and Xu F 14. The heterogeneity was greatly decreased in subgroup 1 than that in subgroup 2 which implied that the heterogeneity was associated with the larger participant's number. The participant's number larger, the heterogeneity greater. Otherwise, the heterogeneity smaller.

### Bias diagnostics

**Figure 6:**
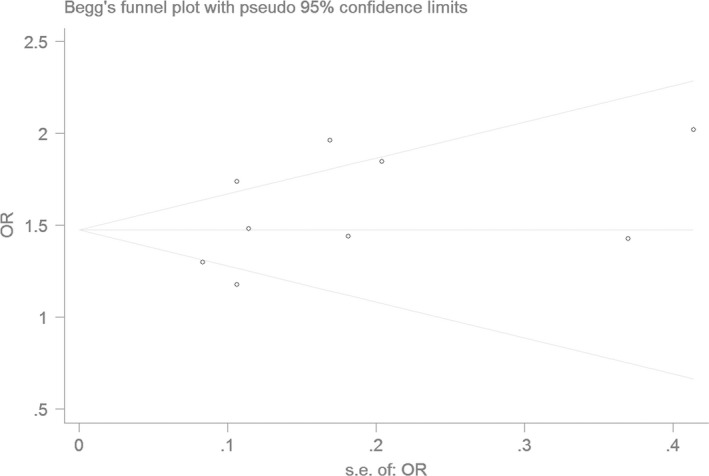
The Begg's funnel plot for studies of the association of CAD associated with *ALDH2* gene G487A polymorphism under an allele genetic model (A *versus* G). The horizontal and vertical axes correspond to the OR and confidence limits. OR: odds ratio

**Figure 7:**
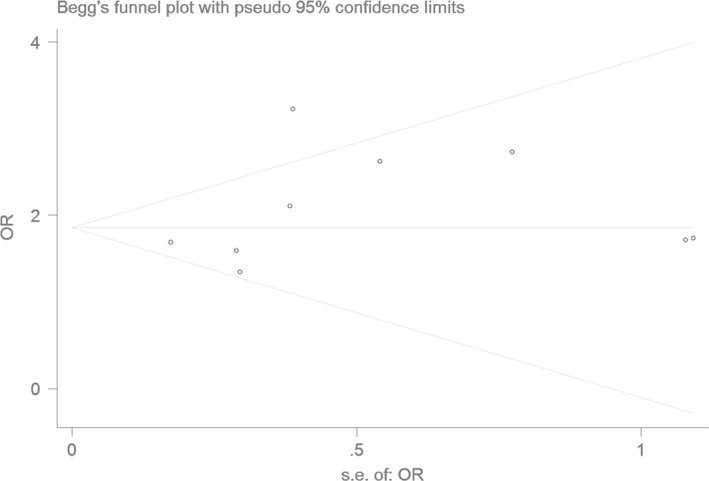
The Begg's funnel plot for studies of the association of CAD associated with *ALDH2* gene G487A polymorphism under a recessive genetic model (AA *versus* GG+GA). The horizontal and vertical axes correspond to the OR and confidence limits. OR: odds ratio

**Figure 8:**
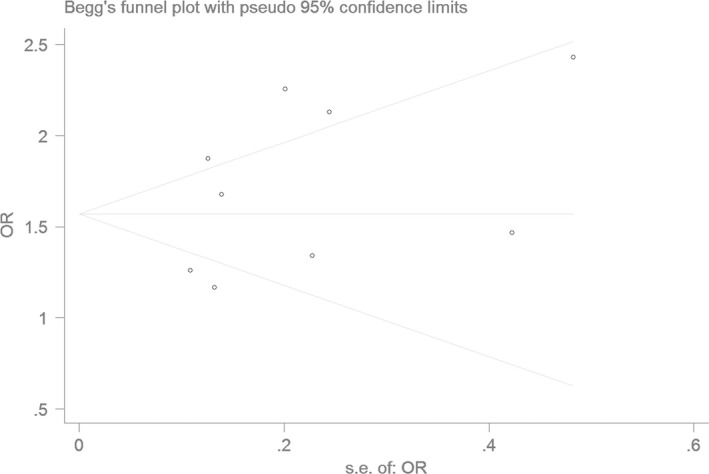
The Begg's funnel plot for studies of the association of CAD associated with *ALDH2* gene G487A polymorphism under a dominant genetic model (AA+GA *versus* GG). The horizontal and vertical axes correspond to the OR and confidence limits. OR: odds ratio

**Figure 9:**
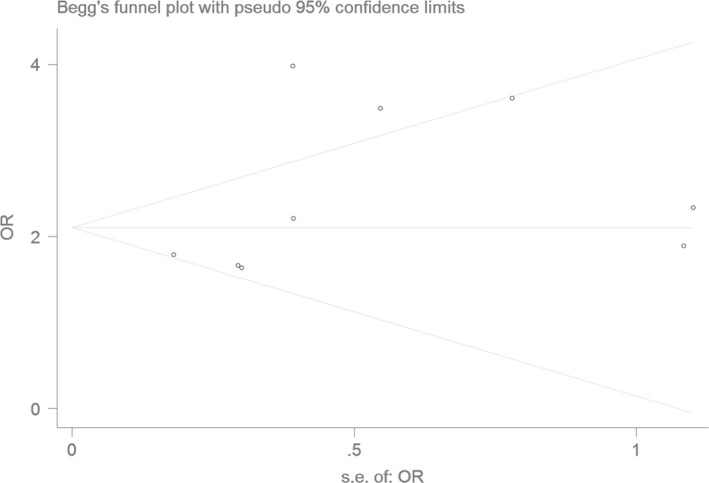
The Begg's funnel plot for studies of the association of CAD associated with *ALDH2* gene G487A polymorphism under a homozygous genetic model (AA *versus* GG). The horizontal and vertical axes correspond to the OR and confidence limits. OR: odds ratio

**Figure 10:**
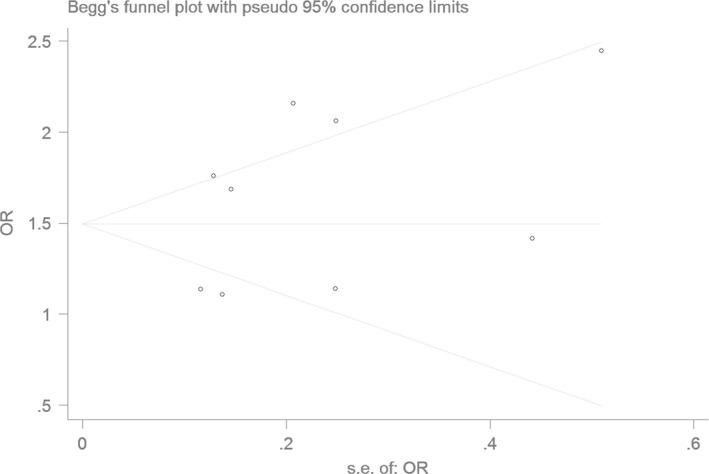
The Begg's funnel plot for studies of the association of CAD associated with *ALDH2* gene G487A polymorphism under a heterozygous genetic model (GA *versus* GG). The horizontal and vertical axes correspond to the OR and confidence limits. OR: odds ratio

Using the funnel plot and Egger's test, the publication bias among the individual studies was assessed. No publication bias was found in the Begg's funnel plot under all of the genetic models (Figs 6, 7, 8, 9, 10). Additionally, no significant difference was found in the Egger's test yet, which indicated that there was no publication bias in the current meta‐analysis under the all of the genetic model (allele: *T* = 1.43, *P* = 0.197; recessive: *T* = 1.21, *P* = 0.266; dominant: *T* = 1.26, *P* = 0.247; homozygous: *T* = 1.52, *P* = 0.173; heterozygous: *T* = 1.16, *P* = 0.282).

## Discussion

The present meta‐analysis found a significant relationship between *ALDH2* gene G487A polymorphism and CAD under allele (OR: 1.830), recessive (OR: 1.920), dominant (OR: 1.593), homozygous (OR: 2.280) and heterozygous genetic models (OR: 3.330) in the Chinese population. Under the allele, dominant and heterozygous genetic models, GG0, GG1 and GA1 were identified as the main sources of heterogeneity, respectively. In the subgroup analysis stratified by GG0 and GG1, heterogeneity was greatly reduced in the GG0 < 160 and GG1 < 150 subgroups under the allele and dominant genetic models, respectively (*P* > 0.05). Under the heterozygous genetic model, in the GA1 > 100 subgroup, the heterogeneity was significantly increased (*P* < 0.05). This implies that the *ALDH2* gene G487A mutation is an effective genetic risk marker for CAD in the Chinese population with the A allele carriers of *ALDH2* gene G487A polymorphism being particularly susceptible to CAD.

The increased risk of CAD may be due to the reduced ALDH2's ability in alcohol consumption in the *ALDH2* gene G487A mutation people. Narita *et al*. 22 found that moderate drinkers had the lower cardiovascular events, possibly due to modulated levels of HDL‐C, HbAlc and fibrinogen. Chang *et al*. 23 found that as the key enzyme of alcohol metabolism, *ALDH2* gene could influence the individual alcohol consumption and drink frequency. Okamoto *et al*. 24 found that in the Japanese men, the heterozygous GA genotype of *ALDH2* gene carriers had the less capacity for alcohol than the wild homozygous GG genotype, a result that Yu *et al*. 25 replicated in the Chinese Han population. The accumulation of acetaldehyde secondary to the reduced ALDH2 enzymatic activity can cause flushing, tachycardia and nausea more rapidly in an individual with the gene polymorphism and effectively restrict alcohol consumption. With the alcohol capacity and myocardial infarction (MI) morbidity exhibiting a U‐form relationship, the risk of MI in moderate drinkers is lower than that of abstainers and severe drinkers 26. Moderate amounts of alcohol could elevate the serum HDL‐C level and reduce insulin resistance, inflammation and metabolic syndrome morbidity 27. Additionally, in 2017, Zhao *et al*. also performed an updated meta‐analysis on the association of alcohol consumption and CAD mortality, they found that moderate alcohol use was associated with reduced CAD risk 28.

HDL‐C was highly correlated with the MI, stroke, sudden death, the restenosis after percutaneous transluminal coronary angiography (PTCA) and severe premature CAD 29. Researchers have also demonstrated that when 10% reduction in HDL‐C, increased the CAD susceptibility by 13% 6. This is not surprising considering HDL‐C's diverse anti‐atherosclerosis functions. HDL‐C can associate with cells of the vascular wall and reversibly transport redundant cholesterol to the liver where it is converted to cholic acid. HDL‐C can also turnover the endothelial cells dysfunction, stimulate prostacyclin production and inhibit the endothelial cells apoptosis, platelet aggregation, low‐density lipoprotein oxidation and oxidative stress 30.

Both Wang *et al*. 31 and Gu and Li 32 conducted meta‐analyses on the relationship of CAD and *ALDH2* gene G487A polymorphism in the Asian population and got the similar conclusion, but in the two meta‐analyses, only seven studies of the Chinese Han population were included. The individual study by Xue *et al*. in 2007 lacked a control group and so was excluded from the current analysis 21. Nine individual studies of the Chinese Han population were included in the present meta‐analysis so the current meta‐analysis is likely more reasonable than previous ones.

However, our current meta‐analysis still lacks large‐scale or prospective studies on the relationship of *ALDH2* gene G487A polymorphism and CAD. It is well known that the individuals without alcohol consumption have the higher CAD risk than the moderate drinkers 28. In the abstainers, the *ALDH2* gene G487A polymorphism might not have the same role as that in the alcohol users. Furthermore, serum ALDH2 levels can be influenced not only by the *ALDH2* gene G487A polymorphism, but also by environmental factors, such as hypertension, obesity, diabetes mellitus and hyperlipidaemia. As CAD is a polygenic inheritable disease, the micro‐effects of many genes may collectively influence the CAD susceptibility. Polymorphisms in other genes such as *CD14*,* Interleukin‐6, methylenetetrahydrofolate reductase*,* intercellular adhesion molecule‐1*,* ATP‐binding cassette transporter A1, apo A5*,* FgB* and *CETP*,* plasminogen activator inhibitor‐1* may also affect CAD risk 33, 34, 35, 36, 37, 38, 39.

In conclusion, *ALDH2* gene G487A polymorphism was significantly associated with increased CAD risk in the Chinese population. Individuals with the A allele of *ALDH2* gene G487A polymorphism might particularly susceptible to CAD. This conclusion maybe useful in the formulation of a novel personalized approach to CAD treatment. In view of the limitations mentioned above, more studies on the association of *ALDH2* gene G487A polymorphism and CAD are needed to further verify the conclusions in a definite manner.

## Conflict of interest

The authors have no conflict of interest to disclose.

## Supporting information


**Appendix S1.** PRISMA 2009 Checklist.

**Appendix S2.** PRISMA 2009 Flow Diagram.
