# Supplementary material for: ALDH2 gene G487A polymorphism and coronary artery disease: a meta‐analysis including 5644 participants
Source: J Cell Mol Med. 2017 Dec 26;22(3):1666–74. doi: 10.1111/jcmm.13443 (PMC5824379; doi:10.1111/jcmm.13443)
Supplement: Supplementary file 2 — Appendix S2. PRISMA 2009 Flow Diagram. [file JCMM-22-1666-s002.docx]

**PRISMA 2009 Flow Diagram**


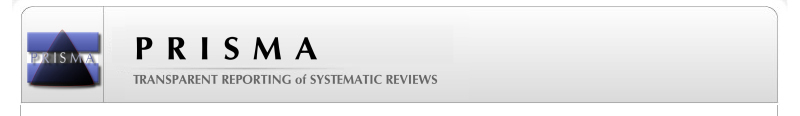


Records excluded for lacking control group (n = 1)

Studies included in qualitative synthesis
(n =9)

Records excluded for repeated publication
(n = 0 )

Records excluded for no association with *ALDH2* gene G487A polymorphism or CAD

(n =4)

Full-text articles excluded for deviation from HWE (n =1 )

Records excluded for review characteristic
(n =2 )

Articles assessed for eligibility
 (n =10)

Full-text articles assessed for eligibility
(n =14)

Records screened
(n =15 )

Records after duplicates removed
(n =17)

Additional records identified through other sources
(n =0 )

## Identification

## Eligibility

## Included

## Screening

Records identified through database searching
(n =17 )
